# Elastic Deformations in 2D van der waals Heterostructures and their Impact on Optoelectronic Properties: Predictions from a Multiscale Computational Approach

**DOI:** 10.1038/srep10872

**Published:** 2015-06-16

**Authors:** Hemant Kumar, Dequan Er, Liang Dong, Junwen Li, Vivek B. Shenoy

**Affiliations:** 1 Department of Materials Science and Engineering, University of Pennsylvania, Philadelphia, 19104 PA, USA

## Abstract

Recent technological advances in the isolation and transfer of different 2-dimensional (2D) materials have led to renewed interest in stacked Van der Waals (vdW) heterostructures. Interlayer interactions and lattice mismatch between two different monolayers cause elastic strains, which significantly affects their electronic properties. Using a multiscale computational method, we demonstrate that significant in-plane strains and the out-of-plane displacements are introduced in three different bilayer structures, namely graphene-hBN, MoS_2_-WS_2_ and MoSe_2_-WSe_2_, due to interlayer interactions which can cause bandgap change of up to ~300 meV. Furthermore, the magnitude of the elastic deformations can be controlled by changing the relative rotation angle between two layers. Magnitude of the out-of-plane displacements in graphene agrees well with those observed in experiments and can explain the experimentally observed bandgap opening in graphene. Upon increasing the relative rotation angle between the two lattices from 0° to 10°, the magnitude of the out-of-plane displacements decrease while in-plane strains peaks when the angle is ~6°. For large misorientation angles (>10°), the out-of-plane displacements become negligible. We further predict the deformation fields for MoS_2_-WS_2_ and MoSe_2_-WSe_2_ heterostructures that have been recently synthesized experimentally and estimate the effect of these deformation fields on near-gap states.

2D materials such as graphene, hexagonal boron nitride (h-BN), phosphorene, MoS_2_ and other transition metal dichalcogenides hold promise for a range of electronic and optoelectronic devices such as field-effect transistors, touch-screen panels, energy harvesting devices and ultrafast lasers. Recent technological advancements in the isolation and transfer of different 2D materials without loss of material quality have led to the renewed interest in stacked/layered materials and heterostructures^1^,^2^,^3^,^4^,^5^,^6^. For example, Yu *et al.*^7^ have shown that vertically stacked graphene-MoS_2_-graphene and graphene-MoS_2_-metal junctions can be tuned to obtain photocurrent with quantum efficiency as high as 55%. Similarly, graphene stacked on hBN has been shown to possess electron mobilities that are at least two orders of magnitudes higher compared to graphene on the conventional substrates such as silica or silicon^8^. Other substrate induced phenomenon e.g. band gap opening, Hofstadter butterfly effects and emergence of new Dirac points have also been reported recently^9^,^10^,^11^. However, lattice mismatch and interactions between different layers of a heterostructure can give rise to deformations (both in-plane and out-of plane) of the planar layers, which in-turn lead to changes in electronic properties.

Recently Woods *et al.*^12^ have observed domains of non-uniform strains forming Moiré patterns for small misorientation angles in hBN supported monolayer graphene. These patterns vanish for larger misorientation angles (~15°) between the two lattices, indicating interesting interplay of substrate interactions and mechanical deformations. Similar domains of non-uniform strains due to substrate interactions have also been experimentally reported for other materials e.g. graphene on Ir^13^, graphene on MoS_2_^14^ and MoS_2_ on WS_2_^2^. These deformations often lead to strain dependent changes in electronic properties, in particular, the bandgap. Strains in the graphene on hBN have been shown to open the bandgap^15^,^16^,^17^ in the range of 4 meV to 14 meV. The theoretically predicted^18^ redshift of ~100 meV in the bandgap of MoS_2_ (and higher values for heavier TMDs) per percent of applied strain has been recently confirmed by photoluminescence experiments^19^. Besides the in-plane strains, the out-of-plane displacements may also induce significant changes in the electronic properties of 2D materials. It was shown that out-of-plane displacements with Moiré symmetry in graphene results^20^ in an bandgap opening of 23 meV. For the MoS_2_ bilayer DFT calculations shows that the interlayer coupling varies with the twist angle and distance between two layers which increases the bandgap from 1.2 eV to 1.7 eV when interlayer distance is increased by 1 Å.

It is clear from the above discussion that elastic deformations play a crucial role in determining the electronic properties of vdW heterostructures. However, current theoretical studies to investigate such interlayer interaction induced deformations are limited in their predictive power mainly due to the large mismatch between the length scales of deformations (ten to hundred nanometers) and the length scales accessible to accurate *ab*-*initio* computational methods. Length scales associated with the Moiré patterns require consideration of large system sizes that are currently not possible using *ab-initio* simulations. Molecular dynamics simulation studies are also limited due to the difficulty in developing empirical force fields for many of the new materials. Recent attempts to study deformations of Moiré patterns are based on various poorly controlled approximations, whose validity is difficult to quantify. For example, Neek-Amal *et al.*^20^ and Jung *et al.*^21^ have studied strains in graphene on hBN using approximate theories; while the former study keeps strains due to out-of-plane deformations, the latter only accounts for strain due to the in-plane displacements in graphene. In a general case, both of the contributions will be equally important, so a general theory that applies to any A-B interface is needed to precisely predict the elastic deformations for bilayer heterostructures.

In this article, we develop a general framework to estimate the deformations in heterostructured bilayers with arbitrary misorientation angle using a multi-scale modeling approach, *i.e.*, continuum finite-deformation simulations informed by ab-initio calculations and infer possible consequences of these deformations on the electronic properties of these materials. vdW-corrected DFT simulations can be used to determine the interlayer interaction for any bilayer structures while the continuum model based on the non-linear theory of elastic plates can be used to study the deformation at larger length scales associated with the Moiré patterns. Continuum models have been successfully used to study the micrometer scale deformations such as rippling, scrolling and warping of single and multilayer graphene sheets and nanoribbons^22^,^23^,^24^. To demonstrate the validity of our method, we choose a specific material system, namely graphene on hBN, for which empirical potentials are available. We have compared the deformations predicted by our multi-scale approach with the deformation from all-atom molecular dynamics simulations, with an excellent agreement between these two approaches. Based on the multiscale simulations, we make predictions for other material systems, in particular, 2D heterostructures of transition metal chalcogenides such as MoS_2_, WS_2_ and MoSe_2_ and estimate the change in bandgaps due to these elastic deformations. Furthermore, we also obtain analytical solutions to estimate in-plane strains and the out-of-plane displacements in 2D materials as a function of material and interaction parameters obtained from DFT calculations. These closed-form solutions can serve as a useful guide in determining appropriate material combinations to engineer strain at 2D interfaces.

## Multiscale Approach to Compute Elastic Deformations

**Figure 1:**
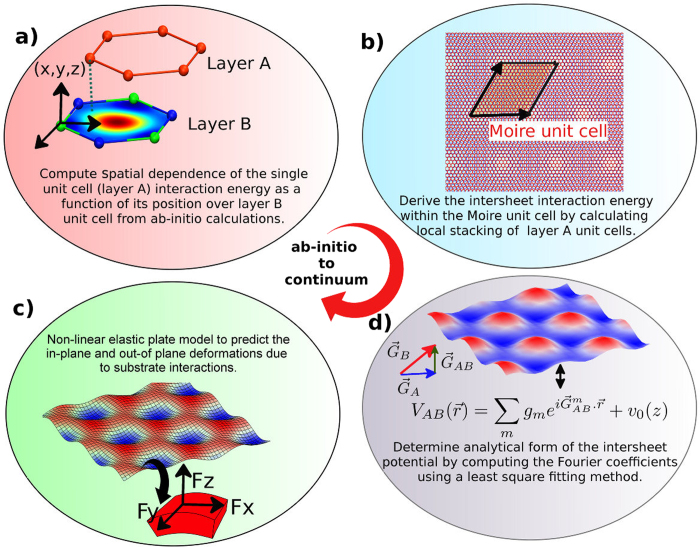
Illustration of the simulation steps. (**a**) Interaction energy of the layer A unit cell as a function of its position over the layer B unit cell computed from the ab-initio calculations. (**b**) Interaction energy of layer A within a Moiré unit cell is determined by calculating the local stacking configuration of the each unit cells in layer A relative to layer B. (**c**) Numerical values of the energy (from b) are used to determine the Fourier coefficients using an inverse Fourier transform. (**d**) Interaction energy is used to compute the in-plane and out-of-plane forces and hence displacements fields in the bilayer using a large deformation elastic plate model.

Our DFT informed continuum approach involves the following steps (as described in Fig. 1) for the case of monolayer of material A (in the present case, graphene) on top of a “substrate” B (in this case, h-BN).

1. Determine the interaction energy of the unit cell of layer A as a function of its position relative to the unit cell of the substrate, layer B.
2. Based on the above interaction energy, derive a continuum description of the spatial variation (on the scale of the Moiré unit cell) of the interactions energy between the layer A and the substrate by inverse Fourier transformation.
3. Obtain in-plane and out-of-plane forces acting on layer A from the spatial variation of the interlayer interaction energy.
4. Use the forces in a non-linear elastic plate model to predict in-plane and out-of-plane deformations in layer A.

Next, we provide the details of the tasks in each step.

Spatial variation of the interaction energy of the unit cell of layer A as a function of its position over the layer B unit cell can be written as (Step 1):
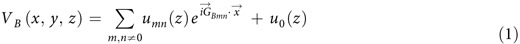
where, G_Bmn_^’s^ are the 2-dimensional reciprocal lattice vectors for the B-lattice. Unknown Fourier coefficients 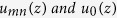 can be computed by calculating the energy of bilayer unit cell in different stacking configurations using density functional theory. For a substrate with triangular lattice symmetry, it is sufficient to consider reciprocal lattice vectors only within the first Brillouin zone. Using this information, Eq. (1) can be written as (Step 1):
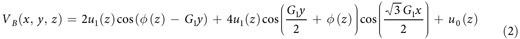
where u_0_(z) is the average energy of the unit cell, u_1_(z) is the magnitude of the energy modulations and ϕ(z) gives the energy difference between AB and BA stacked configurations and G_1_ is the magnitude of the reciprocal vector for substrate primitive unit cell. In the case of Graphene on h-BN, EXX + RPA (exact exchange and random phase approximation) level density functional theory energy calculations were used^15^,^21^ to compute the energy in different stacking configurations. For equilibrium separation z_0_ = 3.4 Å, this gives u_0_ (z) = –69.2 meV, u_1_ (z) = 2.26 meV and ϕ = –50.4°.

Based on this functional form of the interaction energy of the primitive cells, spatial variation of the interaction energy of the two layers within a Moiré cell can be computed by calculating the local stacking of the each unit cell in layer A relative to the unit cell in layer B. Note that since the stacking configurations change only appreciably over the length scales of the Moiré patterns, the variations are gradual on the scale of the lattice spacing. Using this fact, spatial variation of the interaction energy between two layers (see Fig. 1) V_AB_ can be written in terms of Moiré reciprocal lattice vectors 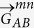 (Step 2):
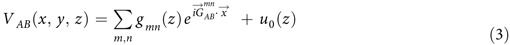


The numerical scheme to relate the unknown Fourier coefficients 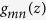 with the coefficients 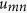 (Eq. 1 for unit cell energy variations) is given in the SI.

Next, we relate the reciprocal lattice vectors of the Moiré cell to the lattice mismatch and the relative rotation of the two layers. For the case of graphene on hBN (with the same lattice symmetry), the lattice mismatch is defined through the a_BN_ = a_c_ (1+δ) and lattice misorientation angle is denoted by θ. Reciprocal lattice vectors 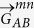 corresponding to this Moiré superlattice are the difference between the reciprocal vector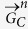 and 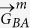.
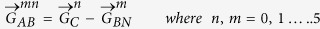
where superscripts m, n denotes one of the six reciprocal vectors for both the lattices given as:
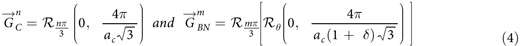
where 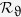 is the two-dimensional rotation operator. For the deformations varying on the scale of the Moiré lattice, it is enough to consider only reciprocal vectors with m = n. Hence the Moiré reciprocal lattice vectors are:
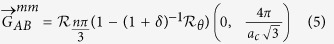
Therefore, interaction energy function for the bilayers with hexagon symmetry can be written as (Step 2):
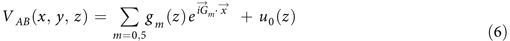
where we have replaced 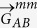 by 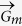 for brevity.

All the components of the force acting on layer A can be computed by taking the derivatives of interaction energies computed in the previous step. However, this will require the knowledge of the Fourier coefficients 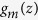 for all interlayer separations. For small deformations, as in the present case, interaction energy can be expanded around the equilibrium separation z_0_ between the layers (Step 2)
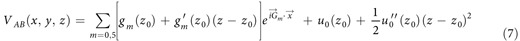
noting that the first derivative 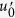 is zero at z_0_ . Here, Fourier coefficient 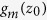 is the magnitude of the energy modulation within Moiré unit cell, 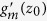 is the change in magnitude of energy modulation and 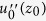 acts as an effective “spring constant” between two layers.

Derivatives of this energy give the components of the forces acting on the top layer due to the substrate (Step 3):
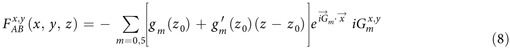

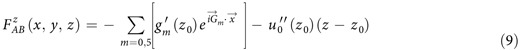
where superscripts x, y, z denotes the vector components.

Since the length scales over which the magnitudes of the forces vary are large (~14 nm) compared to atomic scales, the deformation of the two sheets can be computed using the von-Karman non-linear plate theory^25^. Denoting the out-of plane deflection by w, the equations of mechanical equilibrium are (Step 4):
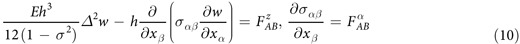
where stress components σ_αβ_ are related to the in plane strains by Hooke’s law:
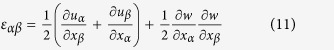
where α, β denote in-plane components x and y, h is the thickness of plate and F’s are from Eqs. (8) and (9). In our calculations, the Young’s modulus E and thickness h of graphene are estimated by equating the effective 2D modulus Eh = E_p_ = 2000 eV/nm^2^ and flexural rigidity to the bending modulus Eh^3^ /12(1– σ^2^) = 1eV. We solve these equations to find in-plane and out-of-plane displacements using the finite element method^24^.

## Results and discussion

For a given bilayer combination, interlayer forces depend both on the magnitude of the lattice mismatch δ and the misorientation angle θ. These forces cause strains in both layers, which are partially relaxed through out-of-plane deformations, depending on the elastic properties of the layers and the nature of interlayer forces. For sake of simplicity we consider the case of layer A on a substrate (for example graphene on h-BN) and present the results for the case of free-standing bilayers in the SI.

### Emergence of Moiré patterns

**Figure 2:**
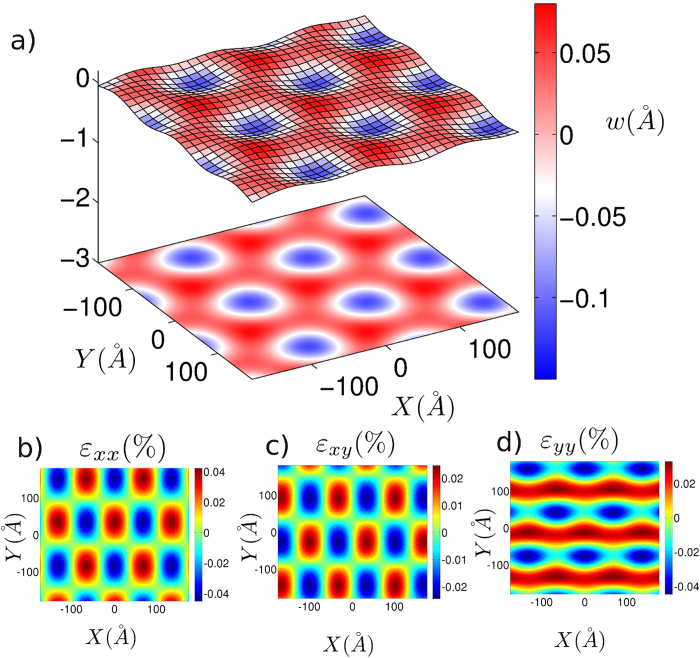
(a) Out-of-plane displacement (w) in the graphene monolayer due to interactions with the hBN substrate for the perfectly aligned layers (θ = 0).We observe Moiré patterns with similar magnitudes as in experiments. Contour plots for the different components of the in-plane strain (**b**) 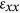 (**c**) 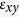 (**d**) 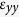.

Due to the forces from the substrate, the graphene monolayer deforms and corrugations develop across the sheet. Our calculations show that the out-of-plane displacements form Moiré patterns as shown in Fig. 2a. Recent experiments have measured^1^ the magnitudes of the out-of-plane displacements to be approximately ~0.3 Å, which is similar to the magnitude of the out-of-plane displacement observed here (0.23 Å). Theoretical calculations shows that such out-of-plane displacements results^20^ in a bandgap opening of ~23 meV in graphene, which is desired for graphene based electronic devices such as high speed transistors. We find that in-plane strains also possess Moiré symmetry as shown in Fig. 2b, c and d. For a graphene sheet perfectly aligned with the hBN substrate, considering the lattice mismatch of 1.8%, the periodicity of Moiré pattern is ~14nm which is consistent with the length of the Moiré pattern observed in different experimental studies^1^,^26^,^27^. Recent experimental studies by Woods *et al.*^12^ have also demonstrated the existence of the hexagonal domains of non-uniform strain in the monolayer graphene on hBN substrate.

Forces arising due to weak vdW interaction between two layers are not strong enough to compensate the lattice mismatch strain; therefore domains of non-uniform strain distribution are created. Strain energy associated with such deformations consists of two parts; one due to curvature (bending) and one due to in-plane strains. In-plane displacements cause lowering of vdW energy by moving to more favorable energy configurations. Out-of-plane displacements are caused by buckling instabilities due to compressive in-plane strains as well as due to different equilibrium interlayer separations for different stacking configurations. Critical strain in each domain is determined by the competition between the penalty associated with the straining the layers and the gain in vdW energy.

### Out-of-plane displacement reduces significantly with increasing angle between two lattices

**Figure 3:**
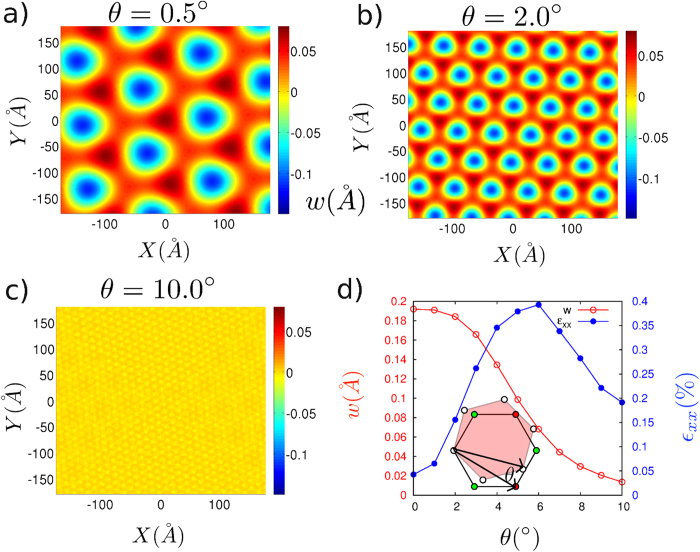
Out-of-plane displacements in the graphene sheet for different misorientation angles θ. Coupling between two sheets decreases with increasing misorientation angles: (**a**) θ = 0.5°, (**b**) θ = 2°, (**c**) θ = 10°, (**d**) Variation of the magnitude of the out-of-plane displacements and in-plane strains for different misorientation angles. Inset shows the definition of the misorientation angle θ.

For a given bilayer with lattice mismatch δ, the period of the Moiré superlattice decreases sharply with the misorientation angle θ between two lattices and is given by:
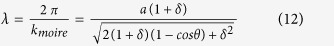


As the cost of bending the graphene monolayer increases with the decrease in length scale over which curvature change appreciably, we find that the magnitudes of the out-of-plane displacements decrease with increase in misorientation angles θ between graphene lattice and hBN lattices (Fig. 3). For the misorientation angle θ ~ 10°, the magnitude of the out-of-plane displacements are almost an order of magnitude smaller as compared to the case of perfectly aligned sheets and no regular pattern is visible (Fig. 3c). Absence of any observable patterns for the shorter Moiré period lengths (~8 nm) is consistent with recent experimental observation by Woods *et al.*^12^ Such rapid decay of the out-of-plane displacements can be quantitatively understood from the analytical solutions we present in the next section.

The in-plane strains show a non-monotonic behavior with increasing misorientation angle. In the Fig. 3d, we plot the strain component 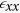 for different misorientation angles between graphene and hBN. For the perfectly aligned case, the magnitude of the out-of-plane displacement is high and hence energy cost of the elastic deformations is dominated by the bending energy. However, with increasing misorientation angle, bending becomes energetically unfavorable and hence, other elastic deformations i.e. membrane strains increase, leading to increased in-plane strains. However, as the misorientation angles increase further, the period of Moiré pattern becomes too small and the cost of straining the lattices does not compensate for the loss in vdW energy and in-plane strains subsequently decrease.

To gain further insight into the interplay between vdW interactions and elastic deformations, we develop an analytical model using elastic plate theory. Our goal is to determine the out-of-plane displacements w(x, y) and in-plane displacements u(x, y) for any given bilayer combination depending on the strength of vdW interactions, material properties and the lattice structure of two sheets. From the numerical solutions, we observe that the out- of-plane displacements do not have a strong dependence on the in-plane forces and similarly the contribution of the out-of-plane displacements to the in-plane strains is small in all cases. In this limit, we can decouple in-plane and out-of-plane displacements and independently determine in-plane displacements as a function of in-plane forces and out-of-plane displacements due to the out-of-plane forces. Governing equations for in-plane displacements are:
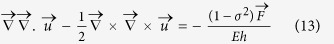
and out-of-plane displacement can be obtained by solving the equation:
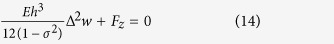
where all vector operators are two-dimensional operators and Δ is the Laplacian operator. All the components of the displacement fields will have the same symmetry as the Moiré lattice and therefore the general form of the displacements are:
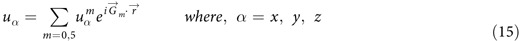


Using this form of the displacements in Eq. (13) and solving for the unknown coefficients u_α_ we get,
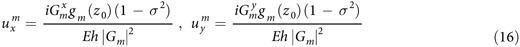


Similarly, Fourier coefficient for the out-of-plane displacement can be obtained from Eq. (14)
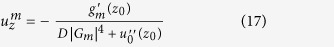
where D is the bending rigidity defined as:


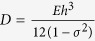


Replacing these Fourier coefficients in the Eq. (15) gives the magnitude as well as spatial variation of the in-plane and out-of-plane displacements in each layer. As shown in Eq. (5), the magnitude of the reciprocal lattice vector G also depends on the relative angle between two lattices and hence this solution gives in-plane and out-of-plane displacements for arbitrary rotation between two sheets. We find that while in-plane displacement coefficients u_x,y_ vary as 1/G, out-of-plane displacement coefficients u_z_ vary as 1/G^4^. This leads to the rapid decay of the magnitude of the out-of-plane displacements with increasing rotation angle between two sheets, as observed in Fig. 3(d). The magnitude of the deformations predicted from theses closed form solutions are in excellent agreement with the results obtained using continuum simulations. Magnitude of the out-of-plane displacements computed from both the approaches for different magnitudes of energy modulation g_m_ have been plotted in Figure S1 (supplementary info).

### Impact of Elastic Deformations on Optoelectronic Properties

**Figure 4:**
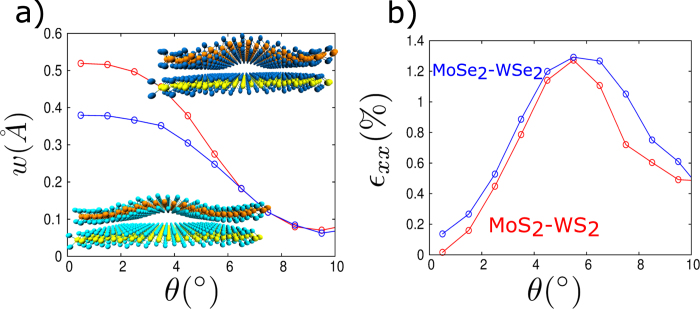
(a) Prediction of the magnitude of the out-of-plane displacement for MoS_2_-WS_2_ and MoSe_2_-WSe_2_ using the multiscale method for different misorientation angles. (**b**) Maximum in-plane strain (ε_xx_) for different misorientation angles between MoS_2_-WS_2_ and MoSe_2_-WSe_2_ bilayers.

Next, we applied the multiscale method on two model systems of vdW TMD heterostructures MoS_2_-WS_2_ and MoSe_2_-WSe_2_ and present in Fig. 4 the magnitudes of the out-of-plane displacements and the in-plane strains for various misorientation angles using continuum simulations with interaction coefficients obtained via vdW-corrected DFT calculations. These TMD heterostructures exhibit similar relationship between the elastic deformations and the misorientation angles as in the graphene-hBN system.

Further insights on how these deformations in these heterostructures affect the electronic and optical properties can be obtained by analyzing the orbital character of the near-gap states. In the monolayer of sulfide of Mo (W), the direct gap opens at *K* point with the conduction band minimum (CBM) derived from 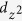 orbital of Mo (W) and the 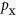 and 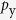 orbitals of S and the valence band maximum (VBM) composed of 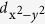 and 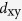 orbitals of Mo (W) and the 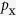 and 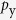 orbitals of S. Because of the weak inter-layer coupling the energy states around *K* point in the MoS_2_-WS_2_ system are nearly a sum of the electronic structures of the individual layers with the VBM in the W layer while the CBM in the Mo layer^28^. The in-plane tensile strain in MoS_2_ layer can induce a decrease of CBM and therefore, a band gap reduction. Our results show that the in-plane strains vary from 0.07% to 1.27% for the MoS_2_-WS_2_ bilayer structure with the misorientation angle changing from 0.5° to 10° and the band gap reduction due to such strains can be as large as ~190 meV (Supporting Information). Similarly, the in-plane strain variations from 0.14% to 1.32% for the MoSe_2_-WSe_2_ bilayer structure can lead to a largest bandgap reduction of ~130 meV (Supporting Information). Non-uniform strain distributions can also influence the bandgap and can be estimated from the pseudomagnetic field generated due to strains^29^. Because of the patterned strain distribution, the excited electrons will be periodically localized around the region with largest in-plane strains. Different from that in TMD heterostructures, the gap tuning in graphene-hBN is mainly due to the interlayer coupling because the states around the Dirac point is derived from the 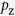 orbitals, which are sensitive to the substrate. In isolated graphene the gap closure is protected by the inversion symmetry. When put on the monolayer h-BN sheet substrate, the non-uniform potential breaks the inversion symmetry and induces a band gap. Although the in-plane strain reserving inversion symmetry cannot open up a band gap, it can effectively tune the Fermi velocity^30^. In our simulations, the strain experienced by the graphene on h-BN sheet can be as large as 0.4% as shown in Fig. 3(d), and the Fermi velocity can be modified by 0.6% compared to the unstrained graphene. As we have shown here, deformations are expected to have a significant effect on the energy states near the Fermi level, and predictions from our method may provide guidance for determining electronic and optical properties of vdW heterostructures.

In addition to the in-plane strains, the out-of-plane displacements may also lead to significant change in the electronic properties. Out-of-plane displacements lead to spatially varying interlayer distances that have been theoretically shown^31^ to cause large modulation in interlayer coupling between MoS_2_ bilayer. Our results show that the magnitudes of the out-of-plane displacements, for the vdW heterostructures studied here, are considerably large (~0.5 Å) and will significantly change the electronic properties. A simple estimate based on MoS_2_ bilayer study^31^ data suggests that an additional bandgap opening of ~200 meV can be achieved with such deformations. Since, the magnitude of the out-of-plane displacements strongly depends on the relative angle between layers, this paves new ways to control the bandgap in these vdW heterostructures. Although such rotation-controlled interlayer coupling has been suggested in earlier studies^11^, only orientation dependent electronic interactions were considered and structural relaxations were ignored. As we have shown here, deformations may have a greater effect on the electronic properties, and predictions from our method will be useful in determining accurate electronic properties of vdW heterostructures.

### Results from the continuum analysis are in excellent agreement with all atom molecular mechanics simulations

**Figure 5:**
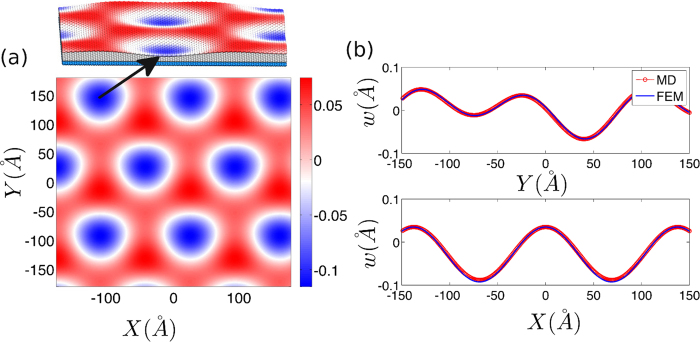
Elastic deformations from molecular dynamics simulations. (**a**) Contour plots of out-of-plane displacements (Å), (**b**) Comparison of the out-of-plane displacements computed from molecular dynamics simulations and continuum simulations. Displacements have been plotted along the armchair (top) and zigzag (bottom) directions.

Molecular mechanics simulations of graphene over hBN substrate were performed using the Adaptive Intermolecular Reactive Empirical Bond Order^32^ (AIREBO) potential as implemented in molecular dynamics package LAMMPS^33^. We consider a graphene sheet of dimensions 42 nm × 42 nm and model the interactions due to hBN using the potential computed from DFT calculations given in Eq. 3. Magnitude of the out-of-plane displacement predicted by molecular mechanics is very close to (0.21 Å) the magnitude obtained from continuum simulations (0.23 Å). In Fig. 5(a) we present the spatial variations of the out-of-plane displacements predicted from molecular mechanics simulations for the perfectly aligned case. Comparison of the out-of-plane displacement values from molecular mechanics, continuum simulation as well as the analytical solution is presented in Table 1. These results further validate our approach to determine the elastic deformation fields by continuum treatment of the graphene sheet.

## Conclusions

1. A multiscale method to compute the elastic deformations for general bilayer structures has been established and validated via comparing the deformations with molecular dynamics simulations as well as with experiments for graphene/hBN bilayer heterostructures. This method is a convenient and precise way to predict the deformation and strains in bilayer 2D heterostructures, and hence paves the way of tuning the electronic and optoelectronic properties of these constructs via strain engineering.
2. Closed form analytical solution to predict the in-plane and out-of-plane displacements are in good agreement with finite element results and remains valid for a vast range of interlayer interactions.
3. Elastic deformation fields for the two newly synthesized vdW heterostructures (MoS_2_-WS_2_ and MoSe_2_-WSe_2_) have also been computed using our multi-scale method. Patterned strains due to these deformations are predicted to induce periodically localized electron states in the upper layer while the hole states remain extended in the substrate layer.
4. The in-plane strains and the out-of-plane displacements due to interactions between bilayers are significant and the near-gap states can be tuned directly by the inter-layer coupling or indirectly through the induced variations in in-plane strains. Tuning the relative angle between two layers can modify the magnitudes of the deformations and hence the electronic and optical properties.

## Additional Information

**How to cite this article**: Kumar, H. *et al.* Elastic Deformations in 2D van der waals Heterostructures and their Impact on Optoelectronic Properties: Predictions from a Multiscale Computational Approach. *Sci. Rep.* **5**, 10872; doi: 10.1038/srep10872 (2015).

## Supplementary Material

Supplementary Information

## Figures and Tables

**Table 1:** Magnitude of the out-of-plane displacements as estimated from different computational approaches for graphene and other 2D materials.

**Method**	**Out-of-plane displacement (Å)**
GR-hBN (AIREBO)	0.21
GR-hBN (Continuum)	0.23
GR-hBN (Analytical)	0.21
MoS_2_-WS_2_ (Continuum)	0.51
MoSe_2_-WSe_2_ (Continuum)	0.39
