# Supplementary material for: Elastic Deformations in 2D van der waals Heterostructures and their Impact on Optoelectronic Properties: Predictions from a Multiscale Computational Approach
Source: Sci Rep. 2015 Jun 16;5:10872. doi: 10.1038/srep10872 (PMC4468582; doi:10.1038/srep10872)
Supplement: Supplementary Information [file srep10872-s1.pdf]

Elastic Deformations in 2D van der waals Heterostructures and Their Impact on Optoelectronic Properties: Predictions From a Multiscale Computational Approach

Hemant Kumar, Dequan Er, Liang Dong, Junwen Li, and Vivek B. Shenoy*

School of Engineering and Applied Sciences, University of Pennsylvania, Philadelphia, Pennsylvania, 19104, USA

E-mail: vshenoy@seas.upenn.edu

General approach to determine interaction energy with Moiré periodicity

The interaction energy of a unit cell in layer A, as a function of its position relative to the unit cell in layer B, can be written as:

$$V_a(x, y, z) = \sum_{m,n} v_{mn}(z) e^{i\vec{G}^B_{mn}\cdot\vec{r}} + v_0(z), \tag{S.1}$$

where coefficients $v_{mn}(z)$ can be computed from the DFT calculations and $\vec{G}^B_{mn}$ are the reciprocal lattice vectors of the primitive unit cell of layer B. Due to small lattice mismatch or mis-orientation angle between two lattices, stacking configurations change appreciably

*To whom correspondence should be addressed

only over the length scales of the Moiré patterns, the spatial variations in the energy are gradual on the scale of the lattice spacing. We use this fact, to write a continuum description of the spatial variation of the interlayer interaction energy using atomic description in Eq. S.1:

$$V_A(x, y, z) = \sum_{m,n} g_{mn}(z) e^{i\vec{G}^M_{mn}.\vec{r}} + v_0(z). \tag{S.2}$$

where $\vec{G}^M_{mn}$ are the reciprocal lattice vectors of Moiré pattern. Note that second term in both of the energy functions are the same, as it is the average energy of a unit cell over all stacking configurations. Below, we discuss how the coefficients (g_{mn}) of the coarse grained energy are related to the coefficients (v_{mn}) of the microscopic interaction energy in Eq. (S.1).

1. Mean interlayer separation z_0 is determined as the separation with minimum average energy:
$$\frac{dv_0(z)}{dz} = 0 \tag{S.3}$$

2. Spatial variations of the interlayer interaction energy within Moiré unit cell are tabulated by assigning the energy to each primitive unit cell according to its local stacking configuration, for a mean interlayer separation z_0.

3. Tabulated values of the interlayer interaction energy are used to determine the unknown Fourier coefficients $g_{mn}(z_0)$ in Eq. (S.2) by taking the inverse Fourier transform:
$$g_{kl}(z_0) = \frac{1}{N_{uc}} \sum_{uc} \left[V_A(x, y, z) - v_0(z_0)\right] \dot{e}^{-i\vec{G}^M_{kl}.\vec{r}}, \tag{S.4}$$
where N_{uc} is the number of primitive unit cells in the Moiré unit cell and sum extends over the all unit cells within Moiré unit cell.

4. Once, we have determined the unknown Fourier coefficients g_{kl}, in-plane spatial variation of the interaction energy can be obtained from Eq. (S.2) for mean interlayer

separation.

Using this information, we determine the forces due to interlayer interactions. For small deformations, energy given by Eq. (S.2) can be expanded around mean spacing z_0:

$$V_A(x,y,z) = \sum_{m,n} \left[g_m(z_0) + g_m^{'}(z_0)(z - z_0) \right] e^{i\vec{G}^M_{mn}.\vec{r}} + v_0(z_0) + \frac{1}{2}v_0^{''}(z_0)(z - z_0)^2 \qquad \text{(S.5)}$$

This spatial variation of the energy leads to the following functional form of the different force components.

$$\vec{F} = -\vec{\nabla} V_A(x,y,z) \qquad \text{(S.6)}$$

$$F_A^x(x,y,z) = -\sum_{m,n} \left[g_{mn}(z_0) + g_{mn}^{'}(z_0)(z - z_0) \right] e^{i\vec{G}^M_{mn}.\vec{r}} i G_{mn}^{M,x} \qquad \text{(S.7)}$$

$$F_A^y(x,y,z) = -\sum_{m,n} \left[g_{mn}(z_0) + g_{mn}^{'}(z_0)(z - z_0) \right] e^{i\vec{G}^M_{mn}.\vec{r}} i G_{mn}^{M,y} \qquad \text{(S.8)}$$

$$F_A^z(x,y,z) = -\sum_{m,n} \left[g_{mn}^{'}(z_0) \right] e^{i\vec{G}^M_{mn}.\vec{r}} - v_0^{''}(z_0)(z - z_0) \qquad \text{(S.9)}$$

If the forces are known, this can be used in the von-karman plate model to predict the deformation of the sheet. While coefficients $g_{mn}(z_0)$ and $v_0(z_0)$ are known, their derivatives can be estimated numerically by repeating the step (2,3,4) for two other separations around the mean separation.

Validity of the analytical solutions

To check the validity of the the analytical solutions, we have compared these solutions with the numerical solutions obtained using finite element simulations for the perfectly aligned graphene-hBN bilayer. Magnitude of the out-of- plane displacement estimated from the finite element simulations is 0.23 Å while analytical solution gives a magnitude of 0.21 Å. Similarly the magnitude of the in-plane displacement in the X direction computed from finite element simulations is 0.0026 Å and the analytical solution results in 0.0023 Å. We

have also checked the validity of these solutions against finite elements results for different interaction parameters. In Figure S1, the magnitude of the out-of-plane displacements computed from both the approaches have been plotted for different magnitudes of energy modulation g_m. It is clear that the closed form solutions remain valid for the interactions that are 20 times stronger as compared to those between graphene/hBN. Only for stronger modulation magnitudes, do the analytical solutions start deviating from the finite element solutions.

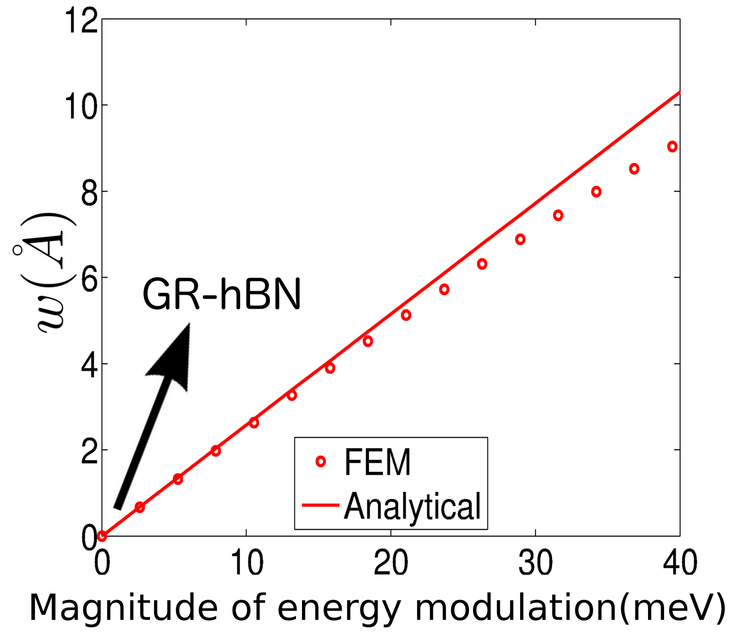

Figure S1: Magnitude of the out-of-plane displacements computed using analytical solutions compared with finite element simulations for different magnitudes of the energy modulation (g_m)

Deformation in Freestanding Bilayer

In case of freestanding bilayer, equal and opposite forces will act on the substrate layer. Due to these forces, deformation can be computed same method described in the main text. Solving Eq. (14,15) for substrate layer gives following

displacement solutions.

$$u_\beta = \sum_{n=0,5} u_\beta^m e^{i\vec{G_m}.\vec{r}} \tag{S.10}$$

Where different displacement coefficients u_β^m are given by:

$$u_x^n = \frac{-iG_n^x g_n(z_0)(1-\sigma_s^2)}{E_s h_s |G_m|^2} \tag{S.11}$$

$$u_y^n = \frac{-iG_n^y g_n(z_0)(1-\sigma_s^2)}{E_s h_s |G_m|^2} \tag{S.12}$$

$$u_z^n = \frac{g_n^{'}(z_0)}{D_s |G_n|^4 - f_0^{"}(z_0)} \tag{S.13}$$

$$D = \frac{E_s h_s^3}{12(1-\sigma_s^2)} \tag{S.14}$$

Where subscript s denotes elastic constant corresponding to substrate layer. E_s is Young's modulus, σ_s is the Poission's ration and h_s is the thickness of the substrate layer.

Interaction energy for $\mathbf{MoS_2 - WS_2}$ and $\mathbf{MoSe_2 - WSe_2}$ unit cells

Interaction energy between these bilayer structures can also be defined using Eq. S.1. The interaction coefficients between bilayer structures of transition metal chalcogenides ($MoS_2 - WS_2$ and $MoSe_2 - WSe_2$) are obtained through first principles calculations based on density functional theory (DFT) as implemented in the Vienna ab-initio simulation (VASP) code [1]. Projector augmented wave (PAW) pseudo-potentials [2] are used with a cutoff energy of 400 eV for plane-wave expansions. The exchange-correlation functional is treated within the Perdew-Burke-Ernzerhof (PBE) generalized gradient approximations (GGA) [3]. The long-rang interlayer van der Waals (vdW) interactions are treated by the newly devel-

oped DFT-TS method [4], wherein the conventional DFT energy is supplemented with a pairwise interatomic vdW potential from non-empirical mean-field electronic structure calculations. The dispersion coefficients and damping function of DFT-TS are charge-density dependent, therefore allowing variations in vdW contributions of atoms due to their local chemical environment. This method agrees well with experiments on the structural parameters of transition metal chalcogenides and many other 2-dimensional materials [5]. For the hexagonal unit cell of the bilayer heterostructures, a Γ-centered k-point mesh of $12 \times 12 \times 1$ in the first Brillouin zone is found to yield well-converged results. A vacuum space of 16 Å thickness is used to prevent any interactions between the adjacent periodic images of the bilayer structures.

First, we determine the lowest energy structures of the $MoS_2 - WS_2$ and $MoSe_2 - WSe_2$

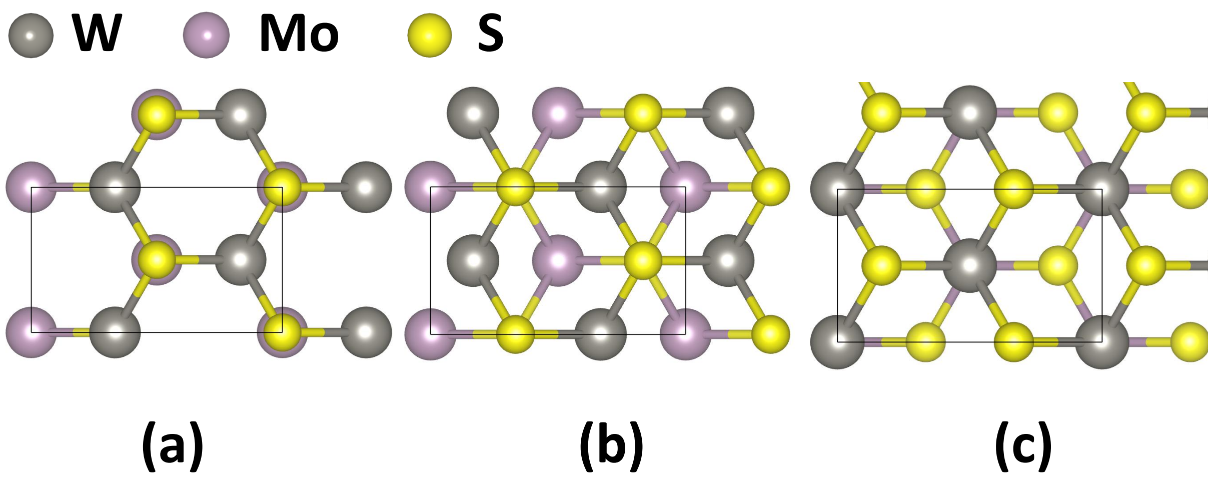

Figure S2: Three high symmetry stacking configurations for which binding energy calculations are done.

heterostructures, which are found to be similar to the 2H stacking in bilayer MoS2 [Fig. S2(a)]. The equilibrium in-plane lattice parameter a of such a stacking and the corresponding Mo-W interlayer distance d are 6.22 Å and 3.16 Å respectively in $\mathbf{MoS_2} - \mathbf{WS_2}$ (6.48 Å and 3.30 Å in $\mathbf{MoSe_2} - \mathbf{WSe_2}$). The atomic positions of this structure are optimized until all components of the forces on each atom are reduced to values below $10^{-5} eV/\AA$. Next, a relative in-plane displacement is introduced to the top layer of a given bilayer structure

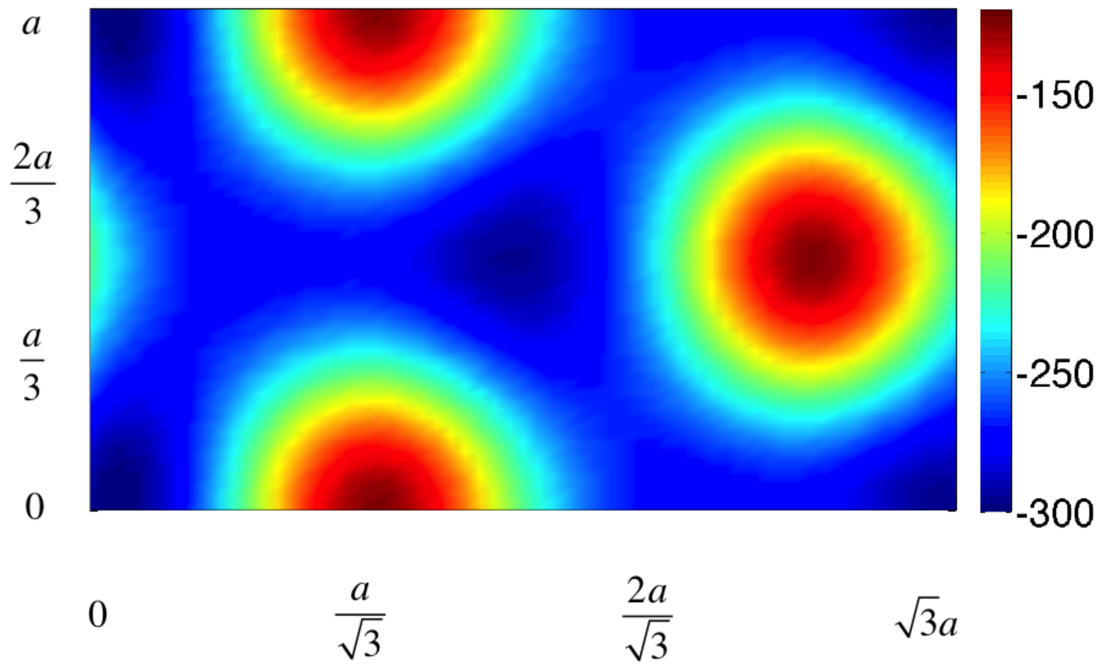

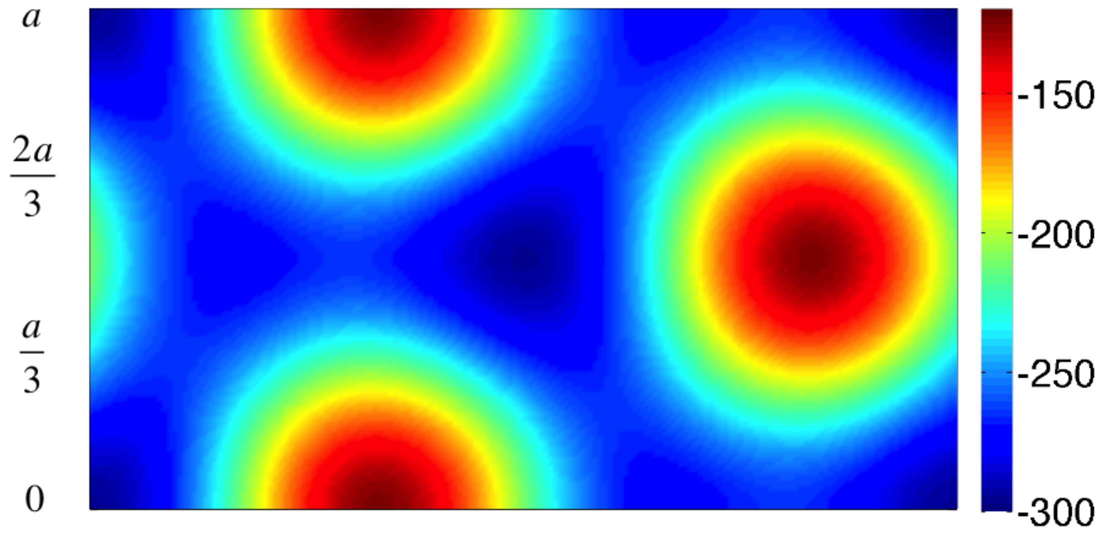

Figure S3: Equivalence of the two approaches to calculate the spatial dependence of the interaction energy. **a**) Interaction energy surface for different stacking configurations computed by energy calculation on 6×6 grid points with unit cell.**b**) Interaction energy surface from the Eq. S.1 where unknown coefficients v_{mn} are determined from the energy values at three high symmetry configurations(AA, AB and BA). Both profiles match very well.

(*e.g.*, $\mathbf{MoS_2 - WS_2}$) while a and d remain unchanged from the equilibrium values. The vdW potential is calculated as a function of such displacements using a 6×6 grid in the rectangular base of the unit cell [Fig. S3(a)]. Two out of the 36 displacements give high symmetric unit cell structures [Fig. S2(b,c)] in addition to the equilibrium structure. The vdW potentials of the three high symmetry structures in Fig. S2 are used to determine the unknown interaction coefficients in Eq(S.1). The coefficients thus obtained are further used to reproduce the vdW potential profile in the rectangular base of the bilayer structure which agrees very well with the potential profile computed by explicit mapping of potential surface [Fig. S3]. Therefore, the obtained interaction coefficients are robust. In other words, just using the vdW potential of these three structures we can generate the energy profile of a bilayer structure with an arbitrary displacement vector. Finally, we repeat the calculations for the three high symmetry configurations with the interlayer distance altered by ± 0.05 Å around the mean interlayer separation distance which is determined by minimum average energy, to compute the z dependence of the different constants in the energy function.

Bandgap modulation due to Deformations

In-plane strains due to interlayer interaction are significant enough to change the bandage of the TMDs monolayers. Uniform strains in $MoS_2 - WS_2$, $MoSe_2 - WSe_2$ heterostructures have been shown[6] to cause bandgap closing of $\sim$ 150 meV and $\sim$ 110 meV respectively for per percent of applied strain. Based on this data, a simple estimate of the bandgap shift for different mosorientation angles have been plotted in the Figure S4, S5. Similarly, our calculation shows that bandgap changes with the interlayer separation. For a given misorientation angle, total change in bandgap was computed by adding both contributions using the following relation.

$$\Delta E_{gap}(\theta) = (\partial E_{gap}/\partial \epsilon)\epsilon(\theta) + (\partial E_{gap}/\partial w)w(\theta) \quad \text{(S.15)}$$

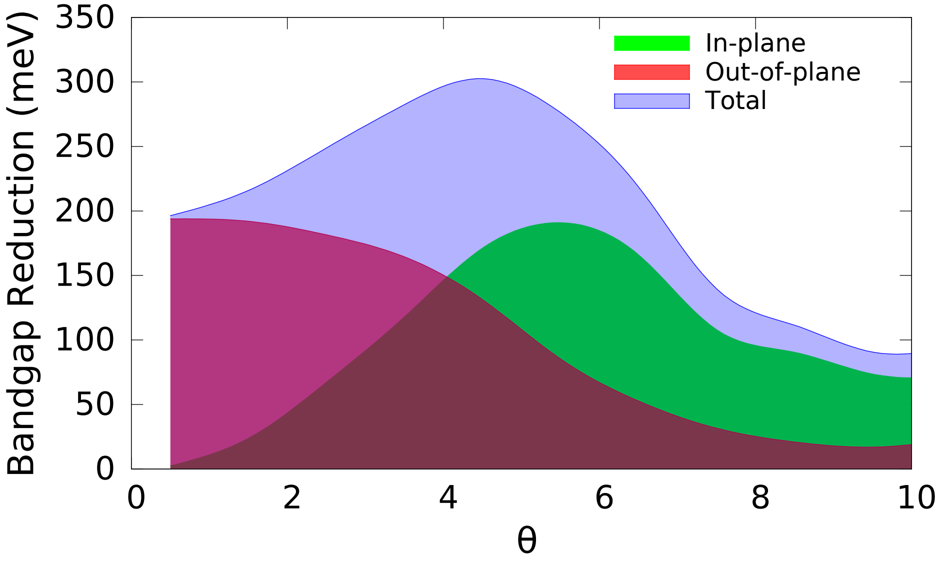

Figure S4: Maximum bandgap reduction in $\mathbf{MoS_2 - WS_2}$ due to in-plane strains and the out-of-plane displacements for different rotation angles. Total bandgap change is calculated by adding both contribution $\Delta E_{gap}(\theta) = (\partial E_{gap}/\partial \epsilon)\epsilon(\theta) + (\partial E_{gap}/\partial w)w(\theta)$

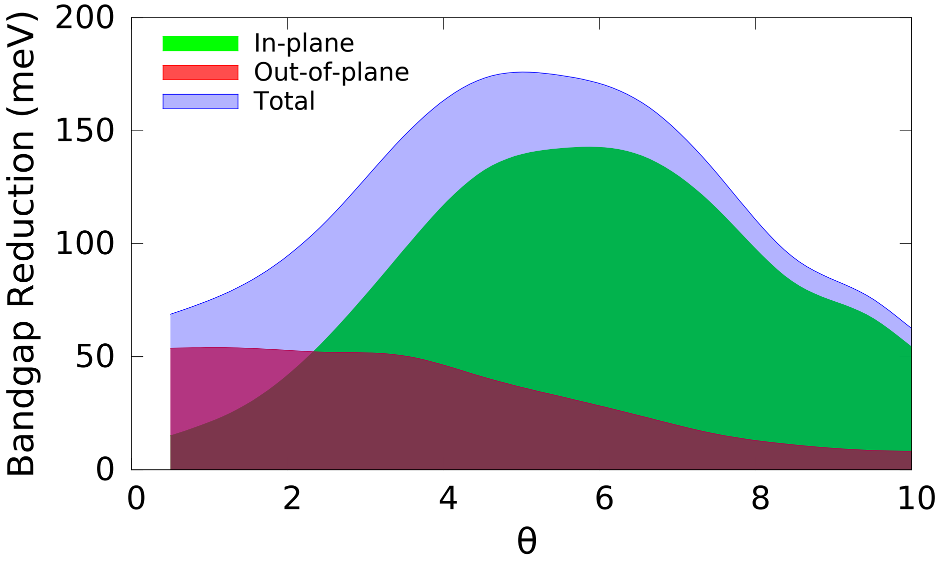

Figure S5: Maximum bandgap reduction in $\mathbf{MoSe_2-WSe_2}$ due to in-plane strains and the out-of-plane displacements for different rotation angles. Total bandgap change is calculated by adding both contribution $\Delta E_{gap}(\theta) = (\partial E_{gap}/\partial \epsilon)\epsilon(\theta) + (\partial E_{gap}/\partial w)w(\theta)$

References

References

[1] G. Kresse and J. Furthmuller, Phys. Rev. B 54, 11169 (1996).

[2] P. E. Blochl, Phys. Rev. B 50, 17953 (1994).

[3] J. P. Perdew, K. Burke, and M. Ernzerhof, Phys. Rev. Lett. 77, 3865 (1996).

[4] A. Tkatchenko and M. Scheffler, Phys. Rev. Lett. 102, 073005 (2009).

[5] T. Bucko, S. Lebegue, J. Hafner, and J. G. Angyan, Phys. Rev. B. 87, 064110 (2013).

[6] M. Sharma, A. kumar, P. K. Ahuluwalia and R. Pandey, APL,116, 063711 (2014).
